# The One Health approach to identify knowledge, attitudes and practices that affect community involvement in the control of Rift Valley fever outbreaks

**DOI:** 10.1371/journal.pntd.0005383

**Published:** 2017-02-16

**Authors:** Osama Ahmed Hassan, Hippolyte Affognon, Joacim Rocklöv, Peter Mburu, Rosemary Sang, Clas Ahlm, Magnus Evander

**Affiliations:** 1 Umeå University, Clinical Microbiology, Virology, Umeå, Sweden; 2 Public Health Institute, Khartoum, Sudan; 3 International Centre of Insect Physiology and Ecology, Nairobi, Kenya; 4 International Crops Research Institute for the Semi-Arid Tropics (ICRISAT), Bamako, Mali; 5 Umeå University, Public Health and Clinical Medicine, Epidemiology and Global Health and Umeå Centre for Global Health Research, Umeå, Sweden; 6 Umeå University, Clinical Microbiology, Infectious Diseases, Umeå, Sweden; School of Veterinary Medicine University of California Davis, UNITED STATES

## Abstract

Rift Valley fever (RVF) is a viral mosquito-borne disease with the potential for global expansion, causes hemorrhagic fever, and has a high case fatality rate in young animals and in humans.

Using a cross-sectional community-based study design, we investigated the knowledge, attitudes and practices of people living in small village in Sudan with respect to RVF outbreaks. A special One Health questionnaire was developed to compile data from 235 heads of household concerning their knowledge, attitudes, and practices with regard to controlling RVF. Although the 2007 RVF outbreak in Sudan had negatively affected the participants’ food availability and livestock income, the participants did not fully understand how to identify RVF symptoms and risk factors for both humans and livestock. For example, the participants mistakenly believed that avoiding livestock that had suffered spontaneous abortions was the least important risk factor for RVF. Although the majority noticed an increase in mosquito population during the 2007 RVF outbreak, few used impregnated bed nets as preventive measures. The community was reluctant to notify the authorities about RVF suspicion in livestock, a sentinel for human RVF infection. Almost all the respondents stressed that they would not receive any compensation for their dead livestock if they notified the authorities. In addition, the participants believed that controlling RVF outbreaks was mainly the responsibility of human health authorities rather than veterinary authorities. The majority of the participants were aware that RVF could spread from one region to another within the country. Participants received most their information about RVF from social networks and the mass media, rather than the health system or veterinarians. Because the perceived role of the community in controlling RVF was fragmented, the probability of RVF spread increased.

## Author summary

Rift Valley fever (RVF), is a neglected, emerging, mosquito-borne disease that has caused outbreaks in Africa and the Arabian Peninsula. RVF outbreaks have a severe negative impact on livestock, human health and economy, placing further demands on communities already experiencing high levels of poverty. We believe there is an immediate need to develop new approaches that will tackle the ongoing spread of RVF. One such approach would prioritize outbreak prevention by involving local communities in the surveillance of emerging zoonotic diseases, empowering local communities as agents of change rather than relegating them as passive victims. RVF is a disease with global implication, but it starts at a local level. Therefore, to control zoonotic disease such as RVF, it is important to understand the local communities’ knowledge, attitudes and practices related to RVF. Using a bottom-up perspective, we investigated the factors that keep the local community from participating in the control of RVF outbreaks at the interface between humans, animals, and the environment. By devising acceptable and cost-effective interventions, we believe local communities can be encouraged to be the first line of defense against RVF outbreaks. Furthermore, policies aimed at curtailing RVF outbreaks would benefit from involvement of the local communities.

## Introduction

Global outbreak of zoonotic infections, not only affect human and animal health but also affect food security, and socio-economic stability. To control such outbreaks require local as well regional cooperation. Most zoonotic outbreaks begin and occur in settings where resources are poor and where the outbreak severely affect the local community [1, 2]. These outbreaks spread both within and outside the country of origin, often with devastating consequences [3]. Zoonotic infections originate and spread at the interface between humans, animals, and their environments, making them candidates for the One Health approach to disease control [4–8]. Although international organizations, government authorities, and academic institutions, believe the One Health concept should be a part of a local community’s response to zoonotic infection, the One Health concept is rarely implemented at the community level. It is undeniable that community involvement is crucial in reducing the risk of zoonotic diseases at the interface between animal-human and their ecosystem.

Rift Valley fever (RVF) is a mosquito-borne viral disease affecting both humans and animals. It can be transmitted by mosquito bites or by direct contact with infected animals, their fluids, or products derived from them [9]. The disease in humans varies from a mild influenza-like illness to more severe forms such as hemorrhagic fever, renal failure, encephalitis, retinitis and miscarriage [9, 10]. Because there is no approved human vaccine or treatment available for RVF, RVF poses a major threat to public health [9]. The infection causes so called “abortion storm” in livestock and deadly epidemics in young animals, with severe consequences for local and national economies [11]. RVF is present in Africa, and as of 2000, it has spread to the Arabian Peninsula [12, 13]. Environmental changes, international travel, trade, and the spread of RVF virus (RVFV) and vectors outside of Africa highlights the potential for its global spread [14–17].

Sudan is an agricultural country with diverse ecology and has the second largest livestock population in Africa. Most of the Sudanese population depends on livestock for food and income. Our previous studies of the RVF 2007 outbreak in Sudan found a gap in knowledge regarding the role of the local community [2, 18]. In this study, we used a bottom-up approach to evaluate the knowledge, attitudes and practices that affect how the local agro-pastoralist community in Sudan confronts an RVF outbreak.

## Methods

### Study area, population, and design

**Fig 1:**
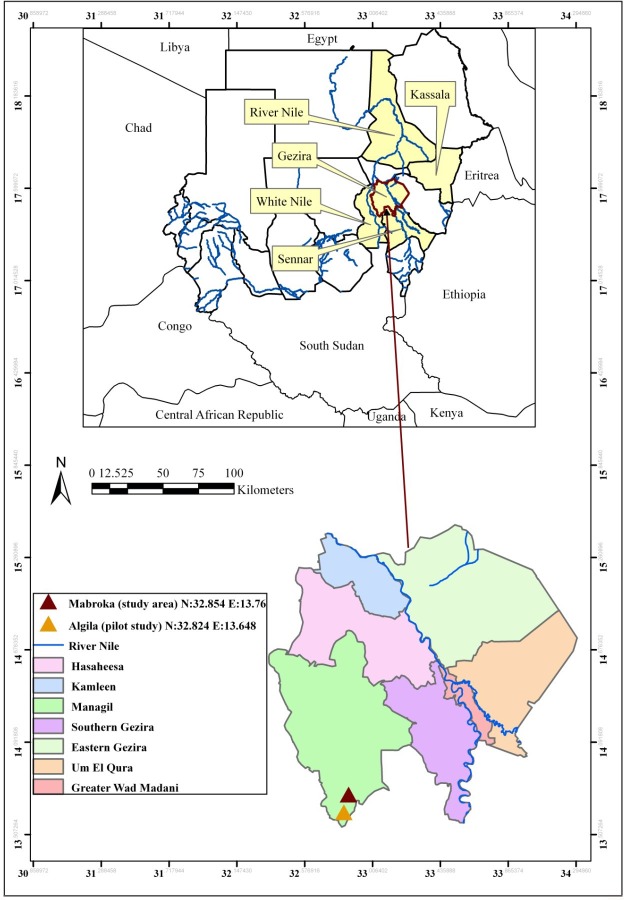
Map of Sudan. The states affected by Rift Valley fever in 2007 (Kassala, River Nile, Gezira, White Nile, and Sennar) are indicated in yellow in the upper map. The pilot study area and the study area in Gezira State are marked with triangles in the lower, expanded map.

In accordance with the guidelines of strengthening and reporting of observational studies in epidemiology (STROBE), a cross-sectional community-based study was conducted in March 2013 at the Mabroka Sagadi village in the Managil locality, Gezira State, Sudan (Fig 1).

The Managil locality is close to irrigation canals. The local people are mainly farmers and shepherds and the locality is home to about 2 million livestock (mainly cattle, sheep and goats). The annual rainfall in the region varies between 100 and 350 mm; however, in 2007 the rain level reached up to 400 mm [19]. The rainy season is from mid-June to September. Managil is located in the Savannah zone and the temperature ranges from 25°C to 46°C.

The study area included all the states affected by the 2007 RVF outbreak (Fig 1). Most of the reported human cases (n = 402) [20] were found in Gezira, so this state was selected. In Gezira, the Managil locality reported the highest number of cases and the village of Mabroka Sagadi had nine recorded human RVF cases during the 2007 outbreak. At the time of the study, there were 5,830 inhabitants in Mabroka Sagadi village. Of the 641 households in the village, 240 households were randomly selected, with a response rate of 98% (n = 235). The household head was defined as the person who in charge of the household and any dependents. We interviewed the household heads and all of them were men or women at least 15 years of age.

### Pilot study and validation of questionnaire

To develop the One Health questionnaire to collect data about RVF at the human-animal-environment interfaces, we intensively searched the literature for information that would help us develop questions regarding the disease and we searched for the best possible answers to these questions supported by the medical literature. This research resulted in the One Health questionnaire that was designed to compile relevant data on RVF in humans, animals, and the environment. Originally written in English, the questionnaire was eventually translated into Arabic (S1 Table). The questions were open ended and the participants were allowed to provide more than one answer. The participants’ answers were compared to the answers listed in the questionnaire but these answers were hidden from the participants to avoid leading questions. If the participant’s answer was not in the listed answers, it was added to the category named “other”.

A two-day training workshop was held for data collectors (public health officers acquainted with the study area) to discuss the objectives of the study, the contents of the questionnaire, and the methods of the study.

A pilot study was conducted in the village of Algila (Fig 1). Algila had similar socio-cultural and ecological characteristics to those of the final study area, and it was also affected by the 2007 RVF outbreak. The findings were analyzed and used to update the questionnaire for the full-scale study.

### Data collection and analysis

A pre-study field visit to the study area was conducted to build trust, explain the study objectives, mobilize the community leaders, and ask the community to be involved in all parts of the study. This led to a sense of ownership and empowerment. Thus, successful face-to- face interviews with the heads of households at their home could take place in a friendly environment.

The thematic areas covered by the One Health questionnaire were socio-demographic considerations, knowledge of RVF in animals and humans, attitudes and practices regarding RVF, and environmental aspects of RVF (S1 Table).

The data were coded, entered into Microsoft Access, and checked for data entry errors by re- entering 10% of the data from the questionnaires. The data were exported and analyzed using STATA version 12 (Stata Corp LP. College Station, TX, USA).

### Ethical statement

Ethical clearance was granted from the Ministry of Health, Gezira State, Sudan. All participants were informed about the objectives of the study and about the confidentiality of the information and results. The participants signed an informed consent document for participation and they were free to leave the study at any time.

## Results

### Socio-demographic characteristics and the effect of RVF on the rural economy

The study included slightly more women (53%) than men (47%), and almost 67% had a low level of education (less than higher secondary school level) with no significant gender difference. Just over half (55%) were above 35 years of age, the vast majority (87%) of the heads of households were married, and their household had at least six members (Table 1). Most women were housewives, while men were mostly occupied with farming (Table 1).

**Table 1:** Socio-demographic characteristics of the study participants.

Category	Subcategory	Number (n = 235)	%
Gender	Male	111	47
	Female	124	53
Age,years	≤ 35	105	45
	> 35	130	55
Educational Level*	Low	158	67
	High	77	33
Occupation**	Housewife,Student,Unemployed	133	57
	Farmer	55	23
	Health professional/Teacher/Freelancer	47	20
Marital status	Unmarried	31	13
	Married	204	87
Breed animals	Yes	170	72
	No	65	28

*Low education is less than higher secondary school.

**The first group was lumped together during the analysis as they were all dependent in that context. The third group was lumped together during the analysis as they were the most educated.

Most of the households bred animals (72%) (Table 1). Cattle were most common, followed by goats and sheep. Animals were kept at home (44%), near the home (22%), or far away from home (6%). Around 25% of the households had members who worked as temporary herdsmen. Most of the households (71%) used their own livestock products as the main source of food, and just under half (43%) sold livestock as a source of income. The 2007 RVF outbreak negatively affected many households (46%), including disrupting the availability of food and livestock trade for about 33% of the households.

### RVF disease, transmission, protection, and sources of information

The awareness about RVF in general was high: 80% of the heads of households heard about the disease. About 9% of the heads of households had seen people who had contracted RVF during the 2007 outbreak. More than half of those interviewed stated that RVF is a zoonosis that affects both animals and humans, and that RVF had been a serious health problem in the area during the 2007 outbreak. The most common sources of information in the community about an RVF outbreak was their social networks of relatives and friends (54%) and mass media (23%); a less common source of information was the health system (6%) or others such as veterinarians (5%).

According to the respondents, there was higher livestock mortality in the area during the 2007 RVF outbreak than the year before (2006) and the year after (2008) the outbreak. During the 2007 RVF outbreak, 34% of the heads of households had experienced death of livestock; in 2006 and 2008 only 17% had experienced death of livestock. This difference, however, could not be confirmed from official reports.

Although the community experienced higher livestock mortality, only some mentioned known symptoms in livestock that died, such as nasal and ocular discharge and hemorrhagic diarrhea (Table 2). Regarding sick livestock, known RVF symptoms were not mentioned by the majority: only 20% stated hemorrhaging and less than 9% mentioned fever and refusal to eat (Table 2). Likewise, RVF symptoms in humans were not well known to the majority of the respondents: 25% stated that fever and hemorrhagic symptoms were most common.

**Table 2:** Perceived RVF symptoms reported by participants regarding their livestock or livestock in the area suspected of having RVF during the 2007 outbreak (n = 235).

	Number of animals	%
**RVF symptoms perceived in animals with fatal disease**		
Nasal and ocular discharge	37	15
Do not remember	17	7
Diarrhea/hemorrhage	12	5
High fever	10	4
Abortion	10	4
**RVF symptoms perceived in sick animals that survived**		
Hemorrhage	47	20
Do not remember	35	15
Fever	19	8
Stopped eating food	18	8
Other mixed symptoms	13	6
Digestive disorders	7	3

**Note**: More than one symptom was allowed.

Most of the respondents did not know how livestock become infected with RVF. For human infection, (21%) stated that humans are infected through uncooked meat while 13% suggested direct contact with livestock. Only a few respondents suggested other mode of transmission such as raw milk (9%), and mosquito bites (6%). The most common answer on how humans should avoid RVF, was to avoid uncooked meat and handling of sick livestock. Avoiding livestock that had suffered miscarriage was the least important according to the results of the survey.

Although few thought that RVF is a contagious disease, more than half of the respondents expected to get RVF when it was present in their area―either due to animal-to-human contact or human-to-human contact (Table 3). One-quarter said that they would avoid contact with neighbors who they suspected of having RVF. About half of the participants (45%) were in favor of patient isolation as a preventive measure during outbreaks and 12% stated that they would avoid contact with RVF patients. Mosquito nets on beds were used by 60% of the respondents, but less than half of these (40%) were impregnated. The majority (60%) had noticed an increase in mosquito population during 2007. To prevent RVF in livestock, the respondents suggested isolation of sick livestock (21%) and vaccination (14%). In addition, the majority of respondents (73%) strongly recommended quarantine of RVF-infected livestock.

**Table 3:** Common perceived reasons as to how the participants could be infected by RVFV if spread in the area compared to what has been reported in the literature.

Percieved reasons	Number of Respondents(n = 235)	%	What has been reported in the literature
Person -to-person contact	94	40	Misconception by participants
We have animals	26	12	Possible if contact with sick animals
I am handling animals	11	5	Possible if animal is sick.
Get it through people coughing	5	2	Misconception by participants
Do not use mosquito bed net	3	1	Possible if bitten by an infected mosquito

**Note**: More than one answer was allowed.

The survey revealed that two-thirds of the participants had a positive attitude about medical treatment against RVF in humans. However, only one-third thought that medical treatment could be used for livestock. They also indicated that veterinarians should diagnose RVF in livestock and health workers should diagnose RVF in human. Around 75% of the heads of households knew where to seek medical treatment, either in the public or the private health sector.

### Reporting and control of an RVF outbreak

If an outbreak occurs, about 40% said they should notify the veterinary authorities about the death of livestock. Almost all the respondents (99%) stressed that they would not get any compensation for their dead livestock if they notified the authorities. When asked if any of their own livestock had had RVF during the 2007 outbreak, 13% said yes. These suspected cases were not confirmed by the authorities. When we asked which disciplines need to work together in order to control RVF, only eight participants (3%) stated that veterinary and health authorities should work together. The majority believed that human health authorities (50%) rather than veterinary authorities (15%) should work to control RVF. With regard to the community’s role in confronting RVF, the respondents suggested that the community should improve its hygienic measures (22%), its health education (18%) and its vector control (8%).

The majority of the study participants (70%) were aware that RVF could spread from one region to another within the country. In addition, 66% of the participants revealed that they were aware of the livestock trade ban associated with RVF outbreak inside and outside the country.

## Discussion

We used the 2007 RVF outbreak in Sudan as a case study to investigate, the knowledge, attitudes and practices, from the One Health perspective that affect the involvement of the local community in disrupting an emerging zoonotic infection. Most of the measures aimed to control RVF were formulated by authorities (i.e., health and veterinary) to be implemented by the local communities (a top-down). For RVF, it seems as these actions are not enough as disease emergence continues. We believed that insights from the local community (a bottom-up approach) about RVF prevention and control could help stop the spread of RVF outbreaks. This bottom-up approach could be a tool to better combat the transmission and the spread of RVF in the regions where the outbreaks are initiated, and could enhance a top down approach. To identify the important factors we used the unique One Health approach to gather information about livestock, people, and the environment. We also investigated whether the community considered the integration of the One Health approach of health, veterinarians, and environment authorities as the best strategy to confront RVF and how they could contribute to RVF control.

Although the community studied had experienced a large outbreak, few had proper knowledge of RVF regarding cause, mode of transmission, symptoms, prevention, and control. This lack of knowledge increases the chances of RVF spreading to neighboring areas and would prevent the community from confronting the disease. The knowledge of risk factors for RVF was insufficient, because the community only practiced some of the measures known to prevent RVF [21] (e.g., eating cooked meat and staying away from sick livestock) while exposing themselves to other risks (e.g., exposure to mosquitoes and handling miscarriage livestock). As in most rural areas, these communities in rural Sudan depend on their livestock as a source of food and income, and often breed and raise their livestock inside or near their homes [22]. The majority did not know that handling livestock that had miscarried was a serious risk factor for RVF [23]. This lack of knowledge could pose a significant risk, especially for rural women, as they tend to take care of livestock at home.

Although the community lives near irrigation canals, which serve as a breeding site for mosquito vectors, the majority ignored mosquitoes as a source of infection. The lack of knowledge of risk factors for transmission could explain the high number of cases reported in the 2007 RVF outbreak in Sudan [18]. Like other mosquito-borne diseases, RVF is associated with heavy rains [23], so the authorities could update communities about rain forecasts. This knowledge would encourage a participatory role of local communities in integrated vector control management similar to the malaria control programs that have been established in most countries where malaria is endemic. The infrastructure of such programs such as established vector control management and the use of impregnated mosquitoes bed nets could also help control other types of mosquito-borne viruses in endemic regions[24, 25].

Therefore, human behavior contributes to the disease emergence. To control the spread of RVF, it is essential to understanding how local communities interact with livestock and the environment. We expect that social scientists, who are well equipped to deal with human behavior changes, will be able to find acceptable ways for rural communities to practice better animal husbandry [26].

For control of RVF, we found the main focus in the community was human health and access to regional hospitals, particularly in the rainy season when the roads are difficult to navigate. Notably, the animal dimension to confronting a zoonosis such as RVF was not well understood. To better implement the One Health approach, authorities could work together with communities to prevent and control emerging zoonotic diseases. This is particularly important because the veterinary services might not be able to cover a vast country like Sudan, where the veterinarians are based in the capital of each locality. The veterinarians visit remote villages for vaccination or investigation of suspected cases of abnormal livestock diseases and visits during rainy season are very difficult due to flooded and otherwise impassable roads. In such a context, voluntary animal health workers from the local communities could be trained to identify livestock diseases, including RVF. This work would take place in collaboration with veterinarians, who have an increasingly important role in global health [27]. Similarly, human voluntary health workers could be trained to identify human diseases. These volunteers could be selected in cooperation with community leaders, which would ensure successful collaboration and communication between health care providers and the community. The sustainability of such a system would depend on a rapid response and support from the authorities when needed by the local communities. These suggestions are in keeping with a participatory approach that regards farmers as being effective partners to curb zoonoses [28, 29].

The rural household economy is affected by RVF outbreaks, regarding both food security and disrupted incomes. There were two opposing interests. The community was only interested in interventions to curb the disease that would not result in the culling of livestock without compensation. The absence of a compensation system weakened the motivation to report early cases in livestock to veterinary authority, a requirement if RVF is to be halted before infecting humans. This lack of compensation could be a possible explanation for the delay in reports of RVF in livestock. If RVF had been identified in livestock early, then livestock as well as human RVF outbreaks in Sudan in 2007 and in Kenya in 1997‒1998 might have been prevented [18, 30]. To support the devastated rural economy due to RVF outbreaks [3], a new policy of compensation for culled or dead livestock must be developed. The respondents stressed the importance of safe vaccination at the right time to prevent their livestock from contracting RVF and preventing the spread of RVF to humans, [31]. Normally, RVF vaccination of livestock is not free in Sudan, an expenditure that might impede locals from regularly vaccinating their livestock, bearing in mind that a new episode of RVF might take some years to re-emerge. Therefore, subsidizing vaccinations for emerging zoonotic diseases might encourage farmers to regularly vaccinate their livestock. In general, RVF livestock vaccines are either inactivated or live attenuated [32, 33]. However, the inactivated vaccine needs multiple doses to booster immunity, making it more expensive and more difficult to distribute. Because the vaccine requires multiple shots, establishing immunity requires time and this vulnerability decreases the vaccine’s usefulness during outbreaks. The live attenuated vaccine is administered as a single dose, but it has shown some teratogenic effects that can lead to abortion among inoculated pregnant animals[34, 35]. However, safe vaccine remains the effective way to protect animals and humans [33, 36].

The respondents were aware of the possibility of RVF spreading inside the country, especially through the free mobility livestock grazing system. They also knew about the economic consequences of a ban on livestock trade after an RVF outbreak. This awareness is important to consider when early warning systems are developed to avoid bias in disease surveillance. The community’s main sources of information on RVF were social network and mass media such as radio, not veterinarians or health workers, who were mainly involved in case notification rather than increasing public awareness [37]. The strong dependence on social networks, rather than on medical and veterinary professionals, could increase the risk of misconceptions if the wrong information is spread. Thus, the World Health Organization recommends that during zoonotic outbreaks interdisciplinary teams of health providers, veterinarians and environmentalists, provide main communication with the public [38]. These teams can communicate through social networks and mass media such as radio, which is one of the most common sources of information in remote areas of many countries. This local involvement will empower the community, allowing them to contribute to notification and control of the outbreaks, and lead them to play proactive roles. This cooperation could strengthen the national surveillance system, which depends mostly on passive notification, a system that might overlook health related events in remote areas. Empowering livestock owners is an opportunity to strengthen the surveillance system for zoonoses, including RVF [39].

Although our study was conducted in 2013 in an area that was affected during the 2007 RVF outbreak in Sudan, up-to-date questions about RVF were also asked at the time of the study. For the questions related to the 2007 RVF outbreak, we considered recall bias. Since the 2007 RVF outbreak, no other recorded hemorrhagic fever outbreaks had occurred in this area according to the participants, so the participants would not have mixed the information about RVF with other similar diseases. In addition, the 2007 outbreak was severe, affecting humans, livestock and the economy in a unique way, which made it easier to remember, decreasing the possibility of recall bias.

In general, the results of this survey are generalizable for the agro-pastoralist regions of Sudan due to the similarity of the context as well as for other countries that experience endemic RVF with similar knowledge, attitudes and practices.

## Conclusions

This study addressed the challenges and opportunities of including local communities in controlling RVF outbreaks at the interface between humans, animals, and their environment. The suddenness of the outbreaks, the lack of treatment, the lack of vaccines, and the complex transmission cycle of RVFV highlights the need to increase community participation in disrupting RVF outbreaks. Crucial challenges include improving the knowledge and correcting misconceptions about RVF that result in risky behaviors. However, by empowering rural communities through education and motivating them to recognize cases early, the authorities could be notified and could act accordingly to support the local community. The willingness of the community to participate in curbing RVF outbreaks is an opportunity that can be effectively managed in a bottom-up approach: the more we know about a community’s knowledge, attitudes and practices related to the emergence of RVF, the better we will be embowering local communities with the best information and strategies to prevent the spread of RVF. That is, this bottom-up approach may result in mutually acceptable and cost-effective interventions that can be used to disrupt transmission of RVF in affected communities.

## Supporting information

S1 Checklist (DOC)

S1 Table One Health questionnaire. (DOCX)
