# Supplementary material for: The One Health approach to identify knowledge, attitudes and practices that affect community involvement in the control of Rift Valley fever outbreaks
Source: PLoS Negl Trop Dis. 2017 Feb 16;11(2):e0005383. doi: 10.1371/journal.pntd.0005383 (PMC5332088; doi:10.1371/journal.pntd.0005383)
Supplement: S1 Table — (DOCX) [file pntd.0005383.s002.docx]

**Appendix - One Health questionnaire.**

Visit date: ………… /………… / …………

Interviewer name:

Supervisor name:

**Section (1)-Human and animal socio-demographic characteristics**

1. How old are you?
   1. 15-25
   2. 26-35
   3. 36-45
   4. 46-55
   5. >55
2. What is your gender?
   1. Male
   2. Female
3. What is your marital status?
4. Single
5. Married
6. Divorced
7. Widowed
8. What is the highest level of education you completed?
   1. Illiterate
   2. Religious education
   3. Primary
   4. Intermediate
   5. Higher secondary
   6. Vocational training
   7. University diploma
   8. University bachelor
   9. M.Sc.
   10. Ph.D.
   11. Other, please specifiy
9. What is the highest level of education completed among your household members?
10. Illiterate
11. Religious education
12. Primary
13. Intermediate
14. Higher secondary
15. Vocational training
16. University diploma
17. University bachelor
18. M.Sc.
19. Ph.D.
20. Other, please specify
21. What is your current occupation?
    1. Student
    2. Farmer
    3. Herdsman
    4. Housewife
    5. Teacher
    6. Governmental
    7. Employee
    8. Private sector employee
    9. Freelancer (Grain mill, Labor) Business man (owns a company that employs more than 6 persons)
    10. Professional (Lawyer, Physician, Lab technician, Nurse, Engineer, Accountant, etc.)
    11. Technician (Blacksmith–Welder-etc.)
    12. Unemployed
    13. Other, please specify
22. How many people live in your household?
    1. Less than 3
    2. 3-6
    3. 7-10
    4. More than 10
23. Do you breed animals?
    1. Yes
    2. No, go to question 11
24. Which types of animals?
    1. Sheep
    2. Goat
    3. Cattle
    4. Dog
    5. Poultry
    6. Other, please specify
25. Where do you keep them?
    1. Inside home
    2. Outside near home
    3. Outside a bit far from the home
    4. Other, please specify
26. Does any of your household members work as herdsman?
    1. Yes
    2. No

**Section (2) - Knowledge about the disease in human and animal**

1. Have you ever heard about the disease Rift Valley fever (RVF)?
   1. Yes
   2. No, go to question 87
2. Did any of your family members catch RVF during 2007?
   1. Yes (Cases to be compared with the MoH registers)
   2. No
   3. I do not know
3. From what source had you heard about RVF?
   1. Radio
   2. Television
   3. Physician
   4. Medical assistant
   5. Nurse
   6. RVF patients
   7. Relatives and friends
   8. Newspapers
   9. Magazine
   10. Veteranarian
   11. Other, please specify
4. What is the host range of RVF?
   1. Human (answer question 16-22, then go to 42)
   2. Animals, go to question 23
   3. Both human and animal.
   4. Mosquitoes (go to question 42)
   5. Other, please specify (go to question 42)
5. What is the cause of RVF in human?
   1. Bacteria
   2. Fungal
   3. Virus
   4. Parasite
   5. Don't know
   6. Other, please specify
6. How can a person get RVF?
   1. Direct contact with infected animals
   2. Mosquito bite
   3. By eating uncooked meat.
   4. By drinking raw milk
   5. Do not know
   6. Other, please specify
7. What are the symptoms of RVF in humans?
   1. Digestive disorder if the liver is infected
   2. Breath shortness and pulmonary infections if a lung is infected
   3. Abdominal pain if stomach or intestine is infected
   4. Neurological symptoms and convulsions if brain is infected
   5. Fever and weight loss
   6. Eye inflammation
   7. Abortion in pregnant women
   8. Hemorrhage
   9. Do not know
   10. Others, please specify
8. How can a person avoid infection with RVF?
   1. Vaccination
   2. Cleaning the house
   3. Carefully washing vegetables before eating them
   4. Avoid eating uncooked meat
   5. Slaughter of animals under veterinary supervision
   6. Avoid handling sick animals
   7. Avoid handling aborted animals
   8. Don't know
   9. Other, please specify
9. In your opinion, who is the most suitable person to diagnose RVF in humans?
   1. Yourself
   2. Family and relatives
   3. Religious man
   4. A nurse
   5. Medical assistant
   6. A physician
   7. Other, please specify
10. Does a patient with RVF need medical tests?
    1. Yes
    2. No, go directly to question (23)
    3. Don't know, go directly to question (23)
11. What are the required medical tests to diagnose RVF in human?
    1. X-rays
    2. Ultrasound
    3. Check a blood sample
    4. Do not know
    5. Other, please specify
12. What is the cause of RVF in animals?
    1. Bacteria
    2. Fungal
    3. Virus
    4. Parasite
    5. I do not know
    6. Other, please specify
13. In your opinion, which type of animals are most affected by RVF?
    1. Cow
    2. Sheep
    3. Goat
    4. Camel
    5. Dog
    6. Cat
    7. Donkey
    8. Horse
    9. Other, please specify
14. How can animals get RVF?
    1. Direct contact with infected animals
    2. Mosquito bites
    3. Eating contaminated food
    4. Other, please specify
15. What are the symptoms of RVF in animals?
    1. Digestive disorder if a liver is infected
    2. Breath shortness and pulmonary infections if a lung is infected
    3. Abdominal pain if a stomach or intestine is infected
    4. Neurological symptoms and convulsions if brain is infected
    5. Fever and weight loss
    6. Eye inflammation
    7. Hemorrhage
    8. Abortion in pregnant animals
    9. Stop eating food
    10. I do not know
    11. Others, please specify
16. How can we avoid animals from getting RVF?
    1. Vaccination
    2. Cleaning the animal house
    3. Isolate the sick animal
    4. Slaughter of animals under veterinary supervision
    5. Isolate aborted animal
    6. Medicines from Veterinarian
    7. Do not know
    8. Other, please specify
17. In your opinion, who is the most suitable person to diagnose RVF in animals?
    1. Yourself
    2. Family and relatives
    3. Veterinarian
    4. A veterinary assistant
    5. Medical assistant
    6. A physician
    7. Other, please specify
18. Do the animals with RVF need medical tests?
    1. Yes
    2. No, go to question 31
    3. Do not know, go to question 31
19. What are the required medical tests to diagnose RVF in animals?
    1. X-rays
    2. Ultrasound
    3. Check a blood sample
    4. Do not know
    5. Other, please specify
20. Have you ever noticed abnormal death in your animals or animals in the area during 2007 outbreak?
    1. Yes
    2. No
    3. I heard about that
    4. I do not remember
21. Have you ever noticed abnormal death in animals or animals in the area before 2007 outbreak?
    1. Yes
    2. No
    3. I heard about that
    4. I do not remember
22. Have you ever noticed abnormal death in your animals or animals in the area after the 2007 outbreak?
    1. Yes
    2. No
    3. I heard about that
    4. I do not remember
23. What was the main symptoms of the dead animals if your answer is yes for any of the questions 31 to 33?
    1. High fever
    2. Nasal and ocular discharge
    3. Abortion
    4. Vomiting
    5. Diarrhea and often hemorrhagic
    6. I do not remember
    7. Other, please specify
24. Do you notify the veterinary authority if you have had animals that died abnormally?
    1. Yes
    2. No
25. Do you get compensation if you notify to the veterinary authority about the animals that died abnormally?
    1. Yes
    2. No, go to question 38
    3. I heard about compensation
    4. I do not know, go to question 38
26. Who is compensating you?
    1. Governmental authority
    2. Agricultural insurance company
27. Have you ever noticed abortion in your animals or animals in the area during 2007?
    1. Yes
    2. No
    3. I heard about that
    4. I do not remember
28. Have you ever noticed abortion in your animals or animals in your area before 2007?
    1. Yes
    2. No
    3. I heard about that
    4. I do not remember
29. Have you ever noticed abortion in your animals or animals in your area after 2007?
    1. Yes
    2. No
    3. I heard about that
    4. I do not remember
30. Have your animals ever experienced RVF during the 2007 outbreak?
    1. Yes
    2. No
    3. I do not know
31. In your opinion, does RVF spread more in a certain year season?
    1. Yes
    2. No, go directly to question 44
    3. Do not know, go directly to question 44
32. In your opinion, during which season is RVF more common?
    1. Summer
    2. Winter
    3. Autum
    4. Not restricted to a certain season
    5. Do not know
33. Did you notice any change in the rain level during the year 2007 RVF outbreak?
    1. Yes, increased at that time compared to previous years
    2. Yes, decreased at that time compared to previous years
    3. No, it was the same
    4. I do not know
34. Did you notice any change in the rain level before the year 2007 RVF outbreak?
    1. Yes, increased at that time compared to previous years.
    2. Yes, decreased at that time compared to previous years
    3. No, it was the same.
    4. I do not know.
35. Did you notice any change in the rain level after the year 2007 RVF outbreak?
    1. Yes, increased at that time compared to previous seasons.
    2. Yes, decreased at that time compared to previous years.
    3. No, it was the same.
    4. I do not know.
36. Have you noticed any mosquito swarms during the 2007 RVF outbreak?
    1. Yes
    2. No, go to question 49
    3. I do not know, go to question 49
37. Was that the normal mosquito type that is usually present in your village?
    1. Yes
    2. No
    3. I do not know
38. Do you think RVF can cross from one region to another within the country?
    1. Yes
    2. No
    3. I do not know.
39. Have you ever heard that the disease has spread to other parts of the country?
    1. Yes
    2. No
    3. I do not know
40. Do you think RVF can cross from your country to another country?
    1. Yes
    2. No
    3. I do not know.
41. Could the disease lead to a livestock trade ban between regions in the country?
    1. Yes
    2. No
    3. I do not know.
42. Could the disease lead to a livestock export trade ban?
    1. Yes
    2. No
    3. I do not know.

**Section (2)- Attitude**

1. If you suspect that you have RVF disease, where you would like to go?
   1. Clinic
   2. Health care center
   3. Official hospital
   4. Private hospital
   5. Other, please specify
2. If RVF spreads in the area, do you think you can get the disease?
   1. Yes
   2. No, go to question 57
3. If yes, Why?
   1. We have animals
   2. I am handling animals
   3. I am working as butcher
   4. I do not use mosquito bed net
   5. Person to person contact
   6. I can get it through people coughing
   7. STD
   8. Other, please specify
4. Do you think RVF could be treated in humans?
   1. Yes
   2. No, go to question 62
5. In your opinion, what type of treatment would be appropriate?
   1. Medical treatment
   2. Traditional treatment, go to question 60
   3. Do not know, go to question 62
   4. Other, please specify and go to question 62.
6. Why should a patient with RVF seek medical treatment?
   1. Effective
   2. Not expensive
   3. Treatment place is nearby
   4. Has no side effects
   5. Other, please specify
7. If the answer was "Traditional treatment", what is the type of such treatment?
   1. Religious man
   2. Herbs
   3. Other, please specify
8. In your opinion, why should a patient with RVF seek traditional treatment?
   1. Effective
   2. Not expensive
   3. Treatment place is nearby
   4. Has no side effects
   5. Other, please specify
9. If your neighbor or someone from the village has RVF, how do you react?
   1. Keep dealing normally with neighbor
   2. Avoid contacting
   3. Other, please specify
   4. I do not know
10. Do you think that it is important to medically isolate patients with RVF?
    1. Yes
    2. No
    3. Sometimes
    4. I do not know
11. Do you think that it is important to medically quarantine animals with RVF?
    1. Yes
    2. No
    3. Sometimes
    4. I do not know
12. Do you think agricultural areas are more prone to RVF than the cities?
    1. Yes
    2. No
    3. I do not know
13. In your opinion, what are the necessary disciplines to work together to control RVF?
    1. Veterinary authority
    2. Health authority
    3. Environmental authority
    4. Community
    5. I do not know
    6. Other, please specify
14. In your opinion, what is the role of the community in confronting RVF?
    1. Health education
    2. Destroy the manmade mosquito habitat
    3. Improve hygienic measure at home
    4. Other, please specify
15. In your opinion, how serious health problem is RVF in your area during the 2007 outbreak?
    1. Small
    2. Middle
    3. Big
    4. Seasonal
    5. Do not know
    6. Other, please specify

**Section (3) - Practices**

1. Do you eat uncooked meat?
2. Yes
3. No
4. Do you drink raw milk?
5. Yes
6. No
7. How do you handle a sick animal?
   1. Separate them inside home and treat them by one by the household members
   2. Leave them as they are with other animals
8. Separate them inside home and bring them to the veterinary clinic
9. Other, please specify
10. Do you help animals to deliver?
    1. Yes
    2. No

1. How do you handle aborted animals?
   1. Help them ourselves by hand and without mask
   2. Help them ourselves using gloves and mask
   3. Bring them to the veterinary clinic by hand and without mask.
   4. Bring them to the veterinary clinic using gloves and mask
   5. I leave them without help
   6. Other, please specify
2. How do you handle dead animals?
   1. Bury them
   2. Burn them
   3. Throw them away outside the home.
   4. Throw them outside at the street to be fed on by dogs
   5. Other, please specify
3. Do you buy the meat of animals that are slaughtered in the slaughterhouse under veterinary supervision?
   1. Yes
   2. No
4. Are you slaughtering animals for meat inside the home?
   1. Yes, continuously
   2. Yes, sometimes
   3. No, go to question 79
5. How do you get rid of the carcass waste when slaughtering inside the home?
   1. Bury them
   2. Burn them
   3. Throw them away outside the home.
   4. Throw them outside at the street to be fed on by dogs.
   5. Other, please specify
6. Why don't you take such animals to be slaughtered in the slaughterhouse under veterinary supervision?
   1. There is no slaughterhouse in the area
   2. Not sure that the slaughterhouse is working through all the day
   3. Not sure of continuous availability of a veterinarian to check them
   4. It is expensive
   5. It is far from our area
   6. Other, please specify

**Section (4)- Economic impact and media habits**

1. In general, did you use animal products as main source of food for your family before 2007 RVF outbreak?
   1. Yes
   2. No, go to question 81
   3. Partially
2. Was that disrupted by the RVF outbreak 2007?
   1. Yes
   2. No
3. Did you use animal milk as main source of food for your children before 2007 RVF outbreak?
   1. Yes
   2. No, go to question 73
   3. Partially
4. Did you stop using milk as main source of food for your children during 2007 outbreak?
   1. Yes
   2. No
5. Did you sell animals as source of family income before the 2007 RVF outbreak?
   1. Yes
   2. No, go to question 75
6. Was that trade income disrupted by the 2007 RVF outbreak?
   1. Yes, strongly
   2. Yes, partially
   3. No
7. Do you sell animal products such as milk, margarine or cheese as source of family income before 2007 RVF outbreak?
   1. Yes
   2. No, go to question 87
8. Was that disrupted by 2007 RVF outbreak?
   1. Yes, strongly
   2. Yes, partially
   3. No
9. Do you use mosquito bed net?
   1. Yes
   2. No, go to question 90
10. Are they impregnated?
    1. Yes
    2. No
    3. I do not know
11. Where did you get those mosquito bed nets?
    1. Local authority
    2. NGOs
    3. Market
    4. Other, please specify
12. In your opinion, who is the most reliable person you can get information about RVF from?
    1. Nurse
    2. Medical assistant
    3. Universities students
    4. Physician
    5. Religious man
    6. Other, please specify
13. In your opinion, who is the most reliable media source you can get information about RVF from?
    1. Newspaper
    2. Magazines
    3. Radio
    4. Television
    5. The mosque or church
    6. Other, please specify
14. Do you wish you could get more information about RVF?
    1. Yes
    2. No
